# Peptide Reactivity of Isothiocyanates – Implications for Skin Allergy

**DOI:** 10.1038/srep21203

**Published:** 2016-02-17

**Authors:** Isabella Karlsson, Kristin Samuelsson, David J. Ponting, Margareta Törnqvist, Leopold L. Ilag, Ulrika Nilsson

**Affiliations:** 1 Department of Environmental Science and Analytical Chemistry, Stockholm University, SE-106 91 Stockholm, Sweden; 2 Dermatochemistry and Skin Allergy, Department of Chemistry and Molecular Biology, University of Gothenburg, SE-412 96 Gothenburg, Sweden

## Abstract

Skin allergy is a chronic condition that affects about 20% of the population of the western world. This disease is caused by small reactive compounds, haptens, able to penetrate into the epidermis and modify endogenous proteins, thereby triggering an immunogenic reaction. Phenyl isothiocyanate (PITC) and ethyl isothiocyanate (EITC) have been suggested to be responsible for allergic skin reactions to chloroprene rubber, the main constituent of wetsuits, orthopedic braces, and many types of sports gear. In the present work we have studied the reactivity of the isothiocyanates PITC, EITC, and tetramethylrhodamine-6-isothiocyanate (6-TRITC) toward peptides under aqueous conditions at physiological pH to gain information about the types of immunogenic complexes these compounds may form in the skin. We found that all three compounds reacted quickly with cysteine moieties. For PITC and 6-TRITC the cysteine adducts decomposed over time, while stable adducts with lysine were formed. These experimental findings were verified by DFT calculations. Our results may suggest that the latter are responsible for allergic reactions to isothiocyanates. The initial adduct formation with cysteine residues may still be of great importance as it prevents hydrolysis and facilitates the transport of isothiocyanates into epidermis where they can form stable immunogenic complexes with lysine-containing proteins.

Skin (contact) allergy is a chronic condition that affects as many as 20% of the population in the western world.^1^ Allergic contact dermatitis, which is the clinical manifestation of contact allergy, is classified as a T-lymphocyte mediated type IV hypersensitivity reaction. Contact allergy can be divided into two phases: sensitization and elicitation. Sensitization is the induction phase, which results in an immunological memory. The elicitation phase takes place upon renewed contact with the same compound, which results in an inflammatory reaction. Contact allergy is caused by reactive chemicals (called haptens) of low molecular weight (<1000 g/mol) and appropriate lipophilicity (LogP ~ 2), which are able to penetrate into the epidermis. The haptens themselves are too small to be recognized by the immune system; however, through their reaction with an endogenous protein they have the ability to trigger an allergic response.^2^ The biomacromolecules in our body are rich in nucleophilic residues, such as thiols (-SH) in cysteine and primary amines (-NH_2_) in lysine. Hence, most contact sensitizers are electrophilic compounds that can modify skin proteins by forming covalent bonds with these nucleophilic moieties.

The recommended OECD method for assessing contact sensitizing potency of various compounds is the murine local lymph node assay (LLNA). However, since 2009 testing of cosmetic products and ingredients on animals is banned in the EU and since 2013 marketing of cosmetic products and ingredients that have been tested on animals is also prohibited in the EU. Thus, most companies today use a combination of *in chemico, in silico*, and *in vitro* assays. One method commonly used is the direct peptide reactivity assay (DPRA), which has been validated by the European Center for Validation of Alternative Methods (ECVAM). The DPRA measures the reactivity of possible skin allergens toward two heptapeptides – one with a reactive lysine (Ac-RFAA**K**AA-COOH) and one with a reactive cysteine (Ac-RFAA**C**AA-COOH).^3^,^4^ Usually, a 10-fold excess of the hapten is used and the peptide depletion after 24 h is considered to be an indication of the sensitizing capacity of the compound.^3^

Isothiocyanate (-NCS) is one example of an electrophilic functional group prone to react with both amines and thiols. Phenyl isothiocyanate (PITC) and ethyl isothiocyanate (EITC) (Fig. 1) have been suggested to be responsible for allergic reactions to chloroprene rubber materials, better known to the public as neoprene, a common material in wetsuits, orthopedic braces, and many types of sports gear. PITC is a metabolite from diphenylthiourea^5^ and EITC is both a degradation product and a possible metabolite from diethylthiourea^6^. Both thioureas are rubber chemicals, used as accelerators in the production of chloroprene rubber. When assessed in the murine local lymph node assay (LLNA), PITC was identified as a strong skin sensitizer^5^ and EITC was identified as an extreme skin sensitizer^6^. Another example of an isothiocyanate is tetramethylrhodamine-6-isothiocyanate (6-TRITC) (Fig. 1), a synthetic compound not present in daily life but used as a fluorescent tracer. The fluorescence of 6-TRITC is relatively photostable and robust toward pH changes. In addition, the risk of overlapping emission between 6-TRITC and biological tissue is minimal. These attractive properties make 6-TRITC into an ideal probe in the search for haptenated proteins/peptides in biological samples to gain better understanding of the underlying mechanism of contact allergy.

In the present work we have studied the reactivity of the isothiocyanates PITC, EITC, and 6-TRITC toward different model peptides to gain information about the possible identity of the immunogenic hapten-protein complexes that these compounds may form when entering the skin. We have used the DPRA peptides as well as a hexapeptide (PHCKRM) containing several reactive amino acids. We found that all three isothiocyanates reacted instantly with the amino acid cysteine. However, over time a transfer to lysine or an *N*-terminal proline in PHCKRM was observed for PITC and 6-TRITC. For EITC the cysteine adduct appeared to be more stable and no or very little transfer from cysteine to lysine/proline was observed. To explain the observed difference in reactivity between PITC and EITC they were evaluated *in silico*. The obtained *in chemico* and *in silico* results were compared with *in vivo* data from the murine LLNA. Based on these results, we discuss the role and importance of haptenation of cysteine and lysine in the induction of contact sensitization of the skin.

## Results

### Sensitization studies

When assessed in the murine LLNA, 6-TRITC induced a massive cell proliferation and gave an EC3 value of 0.92 mM (0.040%), which classifies 6-TRITC as an extreme sensitizer.^7^ SI result for each concentration can be found in supporting information (Table S1). This can be compared with PITC which has been shown to be a strong contact sensitizer with an EC3 value of 30 mM (0.40%)^5^ and EITC which has been classified as an extreme sensitizer with an EC3 value of 4.6 mM (0.040%)^6^.

### Assessment of isothiocyanates in a modified Direct Peptide Reactivity Assay (DPRA)

**Figure 2:**
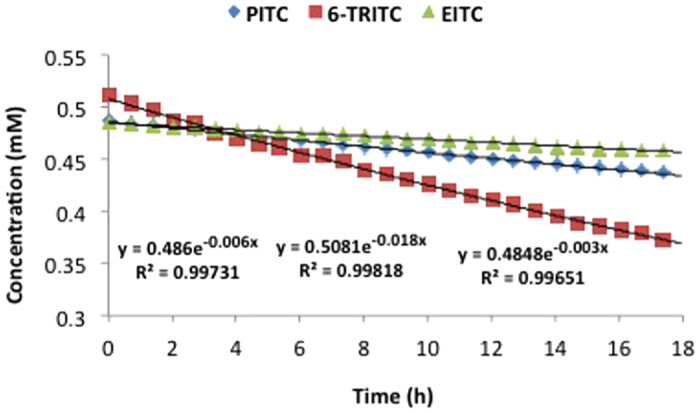
Concentration of the lysine peptide (Ac-RFAA**K**AA-COOH, 0.5 mM) over time in the DPRA with isothiocyanates (5 mM): *blue diamond,* PITC; *green triangle,* EITC; *red square,* 6-TRITC.

Due to instant adduct formation with the isothiocyanates, no curve could be obtained for the depletion of the cysteine peptide in the DPRA (10:1 isothiocyanate/peptide). For 6-TRITC and EITC complete peptide depletion was observed at the first time point (2 min) and for PITC complete depletion was observed at the second time point (42 min). The reactivity of isothiocyanates toward the lysine peptide at pH 7.5 was considerably lower. The lysine adducts were shown to be stable and peptide depletion over time could be measured (Fig. 2). Representative chromatograms and spectra can be found in the supporting information (Figure S1). The most reactive isothiocyanate turned out to be 6-TRITC, which caused a 31% depletion of the lysine peptide after 24 h (Table 1). PITC gave 13% depletion of the lysine peptide and EITC, which was the least reactive of the three isothiocyanates, caused 7.8% depletion after 24 h. It is important to note that the isothiocyanates were not stable under the reaction conditions. In the stability study that was performed only 10–30% remained after 24 h (Figure S2).

Another isothiocyanate, fluorescein isothiocyanate (FITC), has previously been assessed in the DPRA by Gerberick *et al*.^3^ However, as we used different conditions in our lysine peptide depletion assay (ammonium acetate buffer pH 10.2 versus phosphate buffer pH 7.5) we assessed the lysine peptide depletion of FITC under our conditions. FITC gave 27% depletion of the lysine peptide when a tenfold excess of hapten was used (Figure S3).

**Figure 3:**
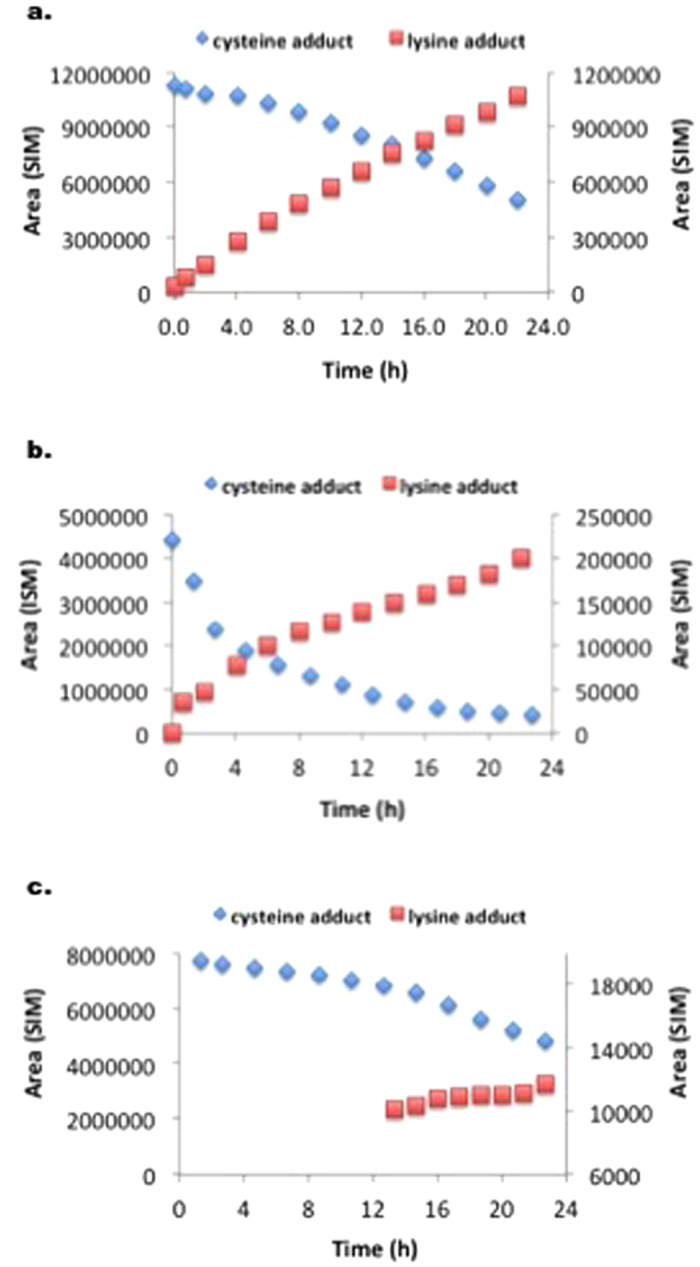
Observed adduct formation in the reactivity experiments with isothiocyanates (0.5 mM), cysteine peptide (0.5 mM), and lysine peptide (0.5 mM). The level of cysteine peptide adduct is decreasing while the lysine peptide adduct concentration is increasing over time: (**a**) 6-TRITC, (**b**) PITC, and (**c**) EITC.

To investigate whether isothiocyanate-moieties can be transferred from the cysteine peptide to the lysine peptide, reactivity experiments were performed with the three isothiocyanates (PITC, EITC, and 6-TRITC) in presence of both the cysteine and the lysine peptide, at equimolar concentrations. For comparison, similar experiments were also performed with either cysteine or lysine peptide. As expected, the isothiocyanates first reacted with the cysteine peptide. However, for PITC and 6-TRITC the isothiocyanate-cysteine adducts decomposed and the isothiocyanate-lysine adduct was formed instead. Interestingly, the EITC-cysteine adduct was more stable and less build-up of the EITC-lysine adduct was detected (Fig. 3).

### Reactivity of isothiocyanates toward the hexapeptide PHCKRM

**Figure 4:**
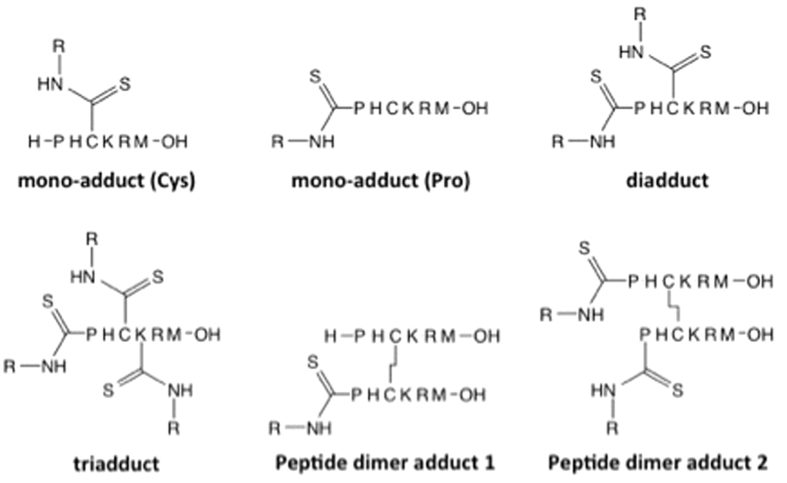
Suggested structures for the detected adducts in the reactivity experiment with isothiocyanates and PHCKRM.

The reactivity of the three isothiocyanates (PITC, EITC, and 6-TRITC) was investigated in another peptide reactivity assay (10:1 isothiocyanate/peptide), developed by Nilsson *et al*.^8^ This assay utilizes a hexapeptide (PHCKRM) with several reactive amino acids. Similar to the DPRA with the lysine peptide experiments, 6-TRITC was the most reactive of the three isothiocyanates. Already at the first time point (2 min) all peptide had reacted and the mono-TRITC cysteine and proline conjugates, respectively, were only minor peaks (Table 2, Figure S4, and Fig. 4), whereas the di-haptenated peptide was the major adduct. After both 12 and 24 h the signal from the tri-haptenated peptide was the most intense.

The reaction with PITC followed the same trend as for 6-TRITC, although proceeding at a lower rate. At the first time point (2 min) small amounts of unreacted PHCKRM could still be detected and the major peak corresponded to a mixture of mono-adducts (Table 2, Figure S5 and Fig. 4). After both 12 and 24 h this had changed and the di-haptenated peptide had become the major product, while both mono-adducts and a tri-adduct were observed as minor peaks.

In the experiment with EITC, a signal from a mono-adduct (Fig. 4) was the predominant (Table 2 and Figure S6) during the entire experiment. The fragmentation pattern (Table S4) reveals that the conjugation site was cysteine. A minor peak corresponding to a di-adduct could be detected after both 12 h and 24 h, while no tri-haptenated peptide could be observed at any time-point.

To find out which amino acid the isothiocyanates have a preference for, the reactivity toward the peptide PHCKRM was also tested at equimolar concentrations. With 6-TRITC, the first observable peaks (2 min) corresponded to a cysteine mono-adduct and a di-haptenated peptide (haptenation of cysteine and proline), respectively (Fig. 4). The latter was observed even when no excess of 6-TRITC was used (Table 3, Figure S7). After 12 h, the concentration of the mono-cysteine adduct had decreased, while instead the intensity of a mono-proline conjugate had increased (Table 3 and Figure S7). After 24 h, the main peak was from a di-haptenated peptide (haptenation of cysteine and proline), while the concentration of mono-adducts had decreased significantly (the cysteine adduct was no longer detectable). The second largest signal corresponded to a cysteine-cysteine peptide dimer with a 6-TRITC on each proline (Fig. 4), according to the fragmentation pattern (Table S5).

**Figure 5:**
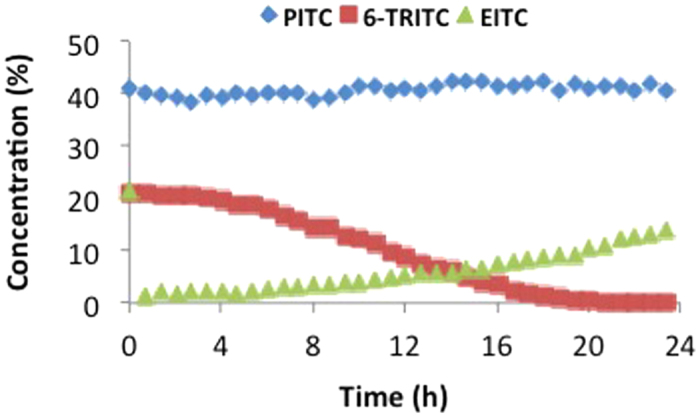
Percentage left of the initial peptide PHCKRM concentration over time in the DPRA with equimolar amounts of isothiocyanates (0.5 mM): *blue diamond,* PITC; *red square,* 6-TRITC; *green triangle,* EITC.

PITC behaved similarly to 6-TRITC, although at a lower reaction rate. More unreacted PHCKRM peptide was left throughout the experiment (Fig. 5). After 12 h a peak corresponding to a di-adduct (haptenation of cysteine and proline) was clearly visible (Table 3 and Figs 4 and S8). However, at the 24 h time-point it had almost completely disappeared. The mono-haptenated peptide gave the highest signal throughout the experiment. The mono-cysteine and mono-proline adducts co-eluted, but the fragmentation pattern (Table S6) showed that at the first time point the peak consisted of mostly the cysteine adduct, but at later time points the proline adduct was the major one. Just as in the 6-TRITC experiment, the second largest adduct peak after 24 h was a haptenated cysteine-cysteine peptide dimer. However, it was a mono-haptenated peptide dimer and not a di-haptenated peptide dimer as for 6-TRITC (Fig. 4).

Although fragments corresponding to a mono-proline adduct were seen, the mono-cysteine adduct was the major adduct at all three time points in the experiment with EITC (Tables 3 and S7, and Figs 4 and S9). Throughout the experiment, the amount of di-adduct (haptenation of cysteine and proline) appeared to be considerably less for EITC compared to 6-TRITC and PITC and the mono-cysteine conjugate seemed to be more stable for EITC than for PITC.

Most PHCKRM depletion after 24 h (100%) was achieved with 6-TRITC (Fig. 5). For PITC approximately 60% of the peptide was depleted already at the first time point, but thereafter the concentration remained more or less constant. EITC on the other hand, gave rise to almost complete peptide depletion at the earlier time-points and showed most peptide depletion of the three isothiocyanates during the first 12 h. However, the formation of the stable amino-adducts appeared to be slower for EITC and as a result the peptide concentration increased slowly with time as the EITC-cysteine adducts decomposed and some of the peptide was regenerated.

### Calculated Reactivity of Isothioyanates toward thiols and amines

**Figure 6:**
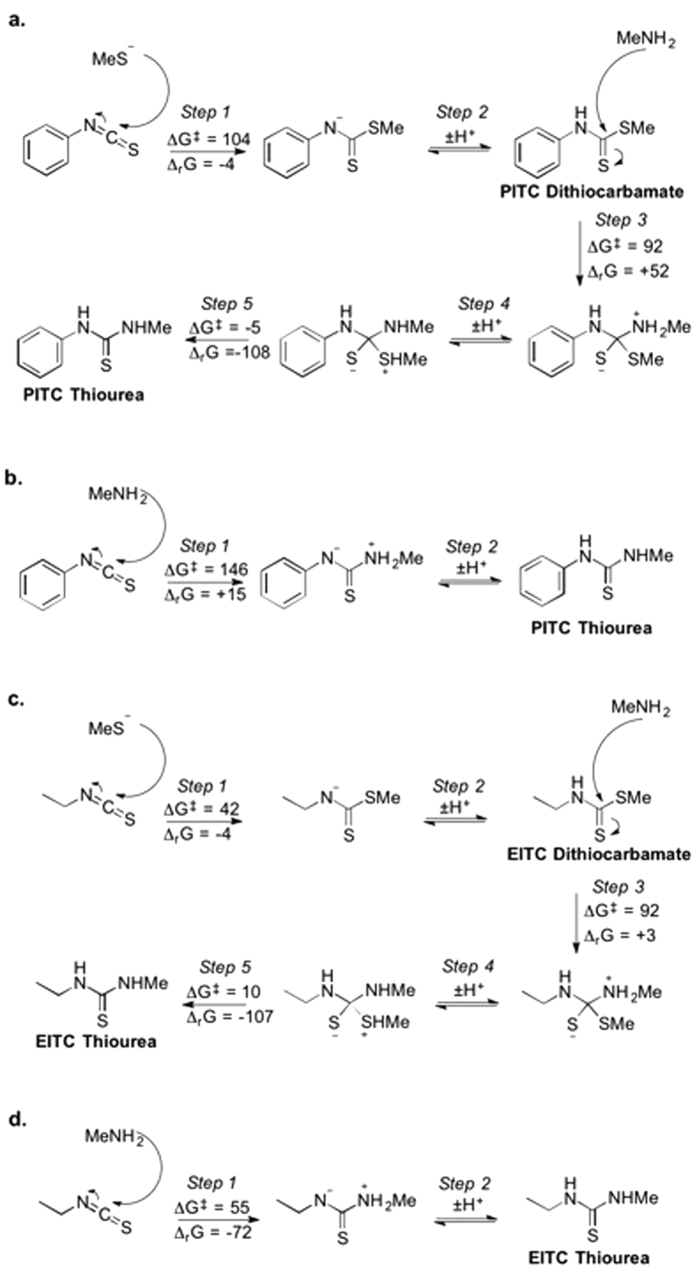
Reactivity parameters, in terms of Gibbs free reaction energy and activation energy, respectively, (Δ_r_G and ΔG^‡^) for the reaction of (**a**) EITC with representative model nucleophiles MeS^−^ followed by MeNH_2_, (**b**) EITC with representative model nucleophile MeNH_2_, (**c**) PITC with representative model nucleophiles MeS^−^ followed by MeNH_2_, (**d**) PITC with representative model nucleophile MeNH_2_. Calculations are performed in Jaguar (B3LYP-D3/6-31+G**). All energies are given in kJ mol^−1^.

The relative energies of the DFT derived transition states (Fig. 6) indicate that, in the case of PITC, further reaction between an amine and a formed dithiocarbamate is preferred (ΔG^‡^ = 92 kJ mol^−1^ in Fig. 6a, step 3, in comparison to 146 kJ mol^−1^ for reaction of the amine with a separate PITC molecule Fig. 6b), whereas in the case of EITC that reaction is less favored (92 kJ mol^−1^ (Fig. 6c, step 3) as opposed to 55 kJ mol^−1^ (Fig. 6d)). The reaction with PITC goes through a high-energy intermediate, which further increases the chance of a cascade of reactions that results in the modification of the proline or lysine since that intermediate is likely to decay. In addition, the second stage (dithiocarbamate to thiourea conversion) has a very rapidly decaying tetrahedral intermediate – indeed is essentially barrierless – in the case of PITC (Fig. 6a, step 5) whereas in the case of EITC this decay is slower and has a (small) energy barrier of 10 kJ mol^−1^ (Fig. 6c, step 5).

## Discussion

Some problems were encountered when performing the DPRA experiments according to the method developed by Gerberick *et al*.^3^ First, DMSO (25% in buffer) gave 100% peptide dimerization within 24 h and could therefore not be used to solubilize the haptens. One of the haptens, 6-TRITC, was not soluble in acetonitrile (ACN), which is the other organic solvent used in the approved method. Instead, methanol (MeOH) was used to solvate all three isothiocyanates, as no substantial peptide dimerization was seen after 24 h when using 25% MeOH in buffer as the solvent system. Second, 6-TRITC was completely degraded within 2 h when ammonium acetate pH 10.2 was used as buffer. The main degradation product is believed to be the corresponding thiourea. Hence, the DPRA with the lysine peptide had to be performed in phosphate buffer pH 7.5, the same as for the cysteine peptide. *These issues highlight the importance of controlling for stability of both hapten and peptide under the conditions used.* As a result of using neutral instead of basic pH, the reactivity of isothiocyanates toward the lysine peptide was considerably slower (due to the higher pKa of lysine) than for the cysteine peptide (Table 1). When the experiments were performed with both peptides simultaneously at equimolar concentrations, the isothiocyanates reacted first with the cysteine peptide. However, the formed cysteine adducts turned out to be unstable for PITC and 6-TRITC and over time the more stable isothiocyanate-lysine adducts were formed instead (Fig. 3a,b). This is in accordance with other studies using aryl isocyanates,^9^ and allyl isothiocyanate^10^. Interestingly, EITC seems to form a more stable cysteine-adduct and as a result less formation of a lysine-adduct was observed (Fig. 3c).

Among the investigated isothiocyanates, 6-TRITC caused most depletion of the lysine peptide (Table 1 and Fig. 2); this is in agreement with the *in vivo* data, as 6-TRITC was found to be the strongest sensitizer of the three isothiocyanates in the murine LLNA. PITC, on the other hand, gave rise to more lysine peptide depletion than EITC (Table 1 and Fig. 2), despite the fact that EITC has been classified as a stronger sensitizer than PITC in the LLNA. Our hypothesis for this phenomenon is either the difference in stability of the two haptens under neutral aqueous conditions, or the difference in stability of the dithiocarbamate adducts that they form with cysteine, or a combination of both. In the present study the PITC-cysteine adduct was found to be considerably less stable than the corresponding EITC-conjugate (Fig. 3), and also PITC itself was shown to be more prone to hydrolysis and alcoholysis. After 24 h in MeOH/phosphate buffer pH 7.5 (1:3), approximately 30% of EITC remained, whereas only 10% was left of PITC (Figure S2).

To be able to understand the observed difference in reactivity and stability between PITC and EITC, computational experiments with methanethiol and methylamine were performed. The relative instability of PITC and the PITC-cysteine adduct can be explained by consideration of the effects on reactivity by the ethyl group of EITC and the phenyl group of PITC. The phenyl group is significantly bulkier, and this destabilizes the tetrahedral intermediate necessary for the reaction between the proline/lysine residue and the dithiocarbamate, rendering this reaction essentially barrierless (Fig. 6a, step 5, ΔG^‡^ = −5 kJ mol^−1^). This therefore pulls the equilibrium between the dithiocarbamate and the intermediate toward the amine-bound product, overcoming the effect on equilibrium of the higher energy (relative to EITC) tetrahedral intermediate (For step 3 in Fig. 6a, ∆_r_G = +52 kJ mol^−1^, compared to +3 kJ mol^−1^ for Fig. 6c), hastening the reaction.

Gerberick *et al*. have suggested a classification model based on DPRA results for prediction of skin sensitization potency of different compounds.^3^ The authors claim that moderate to high reactivity is associated with moderate to strong contact sensitizing potency, and accordingly minimal or low reactivity is associated with weak and non-sensitizing potency. In the suggested classification model an average peptide depletion value after 24 h is calculated using cysteine peptide (10:1 test compound/peptide) and lysine peptide (50:1) experiments. The suggested limits are: <6.376% minimal reactivity, 6.376–22.62% low reactivity, 22.62–42.47% moderate reactivity, and >42.47% high reactivity.^3^ We used the same conditions in our cysteine peptide experiments as were suggested by Gerberick *et al*. However, for the lysine peptide assay there are two differences between the conditions used by these authors and our method. First, they used a higher excess of test compound (50:1 instead of 10:1). Second, they used ammonium acetate buffer at pH 10.2, whereas we used phosphate buffer at pH 7.5. All in all, in comparison to the conditions used by them, our conditions should slow down the depletion rate of lysine considerably. Despite this we obtained an average depletion rate of 54% for EITC, 56% for PITC, and 66% for 6-TRITC. Thus, it should be safe to claim that all three isothiocyanates can be classified as high reactivity compounds in the DPRA. This classification is also in accordance with the *in vivo* data, as all three isothiocyanates have been found to be strong or extreme contact sensitizers in the LLNA. None of the isothiocyanates assessed in the current study had previously been tested in the DPRA. However, FITC, which is structurally similar to 6-TRITC, was one of the compounds used by Gerberick *et al*. in the development of the DPRA classification model.^3^ In the LLNA, FITC was found to be an extreme sensitizer with an EC3-value of 0.14% (3.6 mM), and 15.5% of the lysine peptide was depleted after 24 h in DPRA when a 10-fold excess of FITC was used. As we found that 6-TRITC degraded rapidly in ammonium acetate buffer pH 10.2, we also investigated the stability of FITC under these conditions. We found that FITC, just like 6-TRITC, was completely degraded within 2 h. Therefore, we conducted a peptide depletion experiment with FITC and the lysine peptide (10:1) using the same conditions as for the other isothiocyanates (MeOH/phosphate buffer pH 7.5). FITC gave 27% depletion of the lysine peptide (Figure S3). According to our results, 6-TRITC is a stronger sensitizer (EC3 = 0.92 mM) and the depletion of the lysine peptide is also somewhat larger (31%) for 6-TRITC than FITC. Hence, there is a good correlation between *in vivo* sensitizing capacity and lysine peptide reactivity for these compounds.

By using the peptide PHCKRM we could investigate the reactivity of isothiocyanates toward different amino acids. It has been suggested that the stronger the skin sensitizer the larger the tendency is to modify several nucleophilic sites within the same protein, as this leads to generation of a larger number of different antigens.^11^ Based on this hypothesis, 6-TRITC should be the most allergenic compound, followed by PITC and finally EITC according to the results from the isothiocyanate/PHCKRM (10:1) experiment (Table 2).

From the depletion experiments at equimolar amounts of PHCKRM and isothiocyanate (Fig. 5), it was clear that the three isothiocyanates showed very different reactivity profiles. The presence of unreacted peptide in the experiments with 6-TRITC and PITC, while di-haptenated peptide (and haptenated peptide dimer) was formed, suggests that haptenation of the peptide makes it more reactive (Table 3 and Fig. 5). It is interesting that PITC was shown to be most reactive toward the cysteine site of the free peptide, but as soon as the cysteine-haptenated peptide was formed PITC was transferred to the proline moiety. EITC, on the other hand, appeared to form a much more stable dithiocarbamate as no transfer of EITC from cysteine to proline was observed. This difference in reactivity between EITC and PITC can again be explained using the computational results shown in Fig. 6, which shows that a reaction between a thiol model and EITC is faster (Fig. 6c, step 1, ΔG^‡^ = 42) than the reaction of the dithiocarbamate (cysteine-EITC adduct) with a model amine (Fig. 6c, step 3, ΔG^‡^ = 92). For PITC, on the other hand, the initial reaction of PITC and a model thiol is slower (Fig. 6a, step 1, ΔG^‡^ = 104), than both EITC toward a model thiol (Fig. 6c, step 1, ΔG^‡^ = 42) and than the PITC-dithiocarbamate (cysteine-PITC adduct) toward a model amine (Fig. 6a, step 3, ΔG^‡^ = 92). This is further supported by the experiments with the lysine and cysteine peptide mixture at equimolar amounts where the decrease of PITC-dithiocarbamate (cysteine-peptide adduct) is much faster than the decrease of the EITC-dithiocarbamate. In addition, the formation of the PITC-thiourea (lysine-peptide adduct) is much faster than the formation of EITC-thiourea (Fig. 3).

TRITC appears to fall somewhere in between PITC and EITC. According to the results from the experiment with the cysteine and lysine peptide mixture (Fig. 3), TRITC-dithiocarbamate is more stable than PITC-dithiocarbamate and the decrease of TRITC-dithiocarbamate exhibits similar kinetic as EITC-dithiocarbamate. However, the accumulation of TRITC-thiourea follows a similar formation rate as PITC-thiourea. The equimolar experiments with isothiocyanate-PHCKRM reveal similar trends, i.e. TRITC and TRITC-dithiocarbamate appear both to be more reactive toward amines than EITC, and TRITC-dithiocarbamate is more stable than PITC-dithiocarbamate (Table 3). This would explain why more diadduct is observed for TRITC than for PITC and EITC.

The pyrrolidine functionality of proline has been shown to be a very strong amino-nucleophile.^12^,^13^ It was therefore not surprising to find that haptenation of proline was preferred over haptenation of lysine in the PHCKRM experiments.

There is an ongoing discussion on whether haptenation of cysteine or lysine is most important for the formation of immunogenic hapten-protein complexes that can induce contact sensitization. On one hand, the ability of a compound to react with cysteine has been linked to the development of skin sensitization and is the foundation of KeratinoSens™, another ECVAM approved *in vitro* method developed to replace animal testing.^14^,^15^ On the other hand, in a recent study by Parkinson at al., lysine is found to be much more abundant in the human skin proteome than cysteine.^11^ The same researchers also investigated the amino acid sites of haptenation to human serum albumin for three structurally different contact sensitizers and found that conjugates to lysine and even arginine were far more common than to cysteine, especially after longer incubation times such as two and four weeks.^11^ However, it should be noted that human serum albumin has several reactive lysine residues, but only one reactive cysteine residue. Considering that EITC is a stronger skin sensitizer than PITC *in vivo*, the reactivity toward cysteine appear to be of great importance although the adduct is not stable.

Skin allergy is a delayed T-lymphocyte mediated type IV hypersensitivity reaction, which means that in humans the sensitization phase normally takes 8–15 days and the elicitation phase 48–72 h. Therefore, short-lived hapten-protein complexes that degrade within a couple of hours, such as dithiocarbamates formed from isothiocyanates and cysteine, are unlikely to be able to induce an allergic reaction. Thus, it may indicate that stable thiourea adducts that are formed with lysine are what triggers an allergic reaction toward isothiocyanates.

It has been shown that for both cyanate and benzylisothiocyanate the thiocarbamate adducts that they form upon reaction with cysteine are stable under acidic conditions, but not at neutral or alkaline pH.^16^,^17^ *Stratum corneum* (SC), the top-most layer of epidermis, is acidic with a pH ranging from 4–6. This so called acid mantle works as protection against invading organisms, among other things.^18^ It could be that isothiocyanate haptens, such as EITC and PITC, after skin exposure first react with cysteine moieties in SC in which the dithiocarbamate adducts are stable. The levels of glutathione (GSH), a small cysteine-reactive tripeptide, are high in the upper layer of the skin^19^ and although reactivity with GSH is supposed to be a detoxification process it may have the opposite effect on isothiocyanate haptens. Conjugation with GSH could prevent hydrolysis of EITC and PITC and transport them into viable epidermis where they, due to the increase in pH, could form stable (under physiological pH) hapten-protein complexes with lysines. A study on cutaneous uptake of FITC showed that it accumulated in the SC, probably due to conjugation with proteins in the top-most layer of the skin.^20^ The authors suggested that the reason for the strong sensitizing capacity of FITC could be explained by residing Langerhans cells in the boundary between the viable epidermis and SC that could take up hapten-protein complexes from SC. If that is the case, this could indicate that haptenation of cysteines are of higher importance as stable isothiocyanate-thiol conjugates can be formed in SC due to the lower pH. This would also explain why EITC is more sensitizing than PITC.

To conclude, we show that the isothiocyanates PITC and EITC, believed to be responsible for allergic reactions to chloroprene rubber materials, first react with cysteine-containing peptides. Further, we show that for PITC these adducts are unstable and are transformed into stable lysine adducts over time. It may be that the initial adduct formation with cysteine has two important functions in the induction of contact sensitization. First, it prevents very reactive haptens (under physiological conditions) from degrading. Second, the cysteine-haptenated protein/peptide conjugates enable transport of the haptens into viable epidermis, where they eventually can form stable lysine adducts, thereby inducing contact sensitization and elicitation. EITC, on the other hand, forms more stable cysteine adducts and less lysine adducts are observed. EITC has been found to be a stronger contact sensitizer than PITC in the *in vivo* model LLNA; thus, the formation of stable cysteine adducts may be of more importance than the (slower) formation of lysine adducts.

## Methods

### Caution

Skin contact with EITC, PITC, 6-TRITC, and FITC must be avoided. These are skin-sensitizing substances and must be handled with care.

### Chemicals

MeOH, EITC, FITC, PITC, 6-TRITC, and trifluoroacetic acid (TFA) were purchased from Sigma-Aldrich (Darmstadt, Germany). The cysteine peptide (Ac-RFAA**C**AA-COOH, 97.0%), lysine peptide (Ac-RFAA**K**AA-COOH, 99.0%), and PHCKRM (97.1%) were obtained from Peptide 2.0 (Chantilly, VA, USA). Acetone was from Merck (Darmstadt, Germany), olive oil from Apoteket AB (Göteborg, Sweden), and ACN from Fischer Scientific (Loughborough, UK).

### Instrumentation and modes of analyses

GC/MS analyses were performed for detection of isothiocyanates using electron ionization (70 eV) on a Hewlett-Packard model 5973 mass spectrometer (scanned range *m/z* 50–500), connected to a gas chromatograph (Hewlett-Packard model 6890). The GC was equipped with an on-column inlet and an HP-5MS fused silica capillary column (30 m × 0.25 mM, 0.25 μm film thickness). The column temperature was 100 °C at injection and raised to 200 °C at a rate of 5 °C/min, then raised from 200 °C to 270 °C at a rate of 15 °C/min, and finally held at 270 °C for 20 min.

HPLC/electrospray ionization (ESI)-MS analyses of DPRA mixtures were performed on a Hewlett-Packard 1100 HPLC/MS. The system included a vacuum degasser, a binary pump, an autosampler, a column thermostat, a diode array detector, and a single quadrupole mass spectrometer. The electrospray interface was used with the following spray chamber settings: nebuliser pressure, 35 psig; capillary voltage, 3000 V; drying gas temperature, 350 °C; and drying gas flow rate, 12 L/min. For identification purposes, the mass spectrometer was used in the full scan mode detecting ions from *m/z* 75 to 1500 and with fragmentor voltage of 120 V. For the reactions of isothiocyanates in presence of both cysteine and lysine peptides, the mass spectrometer was used in selected ion monitoring (SIM) mode for 33.3% of the cycle time for two different ions. The remaining 33.3% of the cycle time was used for scan mode (*m/z* 75 to 1500). For 6-TRITC SIM ion 1 (lysine adduct) was set to *m/z* 610.40 ([M+2H]^2+^) and SIM ion 2 (cysteine adduct) was set to *m/z* 597.80 ([M+2H]^2+^). For PITC SIM ion 1 (lysine adduct) was set to *m/z* 456.30 ([M+2H]^2+^) and SIM ion 2 (cysteine adduct) was set to *m/z* 886.40 ([M+H]^+^). For EITC SIM ion 1 (lysine adduct) was set to *m/z* 432.30 ([M+2H]^2+^) and SIM ion 2 (cysteine adduct) was set to *m/z* 838.40 ([M+H]^+^). A Hypersil-Keystone HyPurity C_18_ column (150 mM × 3 mM, 3 μm particles, Thermo Scientific) was used and the column temperature was set to 30 °C. Mobile phase A consisted of 0.1% trifluoroacetic acid (TFA) in milli-Q water, and mobile phase B consisted of 0.085% TFA in ACN. Aliquots of 5 μL were injected onto the column and eluted with a gradient flow of 0.30 mL/min. A linear gradient from 10% B to 90% B (95% B for all EITC experiments) over 30 min was used followed by an 8 min postrun. For the standard curves the areas of the peptide peaks in the 220 nm UV chromatograms were used.

### Sensitization Experiment in Mice

The sensitizing potency of 6-TRITC was investigated using the murine LLNA.^21^ The regional ethics committee, Jordbruksverket, approved the experimental protocol and the method was carried out in accordance with the approved guidelines. The isothiocyanate was tested at five different concentrations using mice in groups of three: 0.00010% (w/v), 0.0010%, 0.010%, 0.10%, and 1.0% (2.3 μM, 23 μM, 230 μM, 2.3 mM, 23 mM). Briefly, groups of female CBA/CA mice received 25 μL of a solution of test compound, dissolved in the vehicle acetone/dibutyl phtalate (1:1 v/v), on the dorsum of the ears daily for three consecutive days. Control animals were treated in the same way with vehicle alone. All mice were injected intravenously 5 days after the first treatment, with 250 μL of PBS (pH 7.4, 137 mM NaCl, 2.7 mM KCl, 10 mM phosphate buffer solution) containing 20 μCi of [^3^H]-methylthymidine. Five hours later, the draining lymph nodes were excised and pooled for each group, and a single cell suspension of lymph node cells was prepared. The thymidine incorporation was measured by β-scintillation counting. Results are expressed as the mean dpm/lymph node for each experimental group and as stimulation index (SI). The stimulation index is defined as the ratio between dpm/lymph node for the test group and the control group. Test materials that at one or more concentrations caused an SI greater than 3 were considered to be positive in the LLNA. EC3 values (the estimated concentration required to induce an SI of 3) were calculated by linear interpolation.^22^ The sensitizing potency of the test compounds was classified according to the following: extreme, ≤0.2%; strong, >0.2% to ≤2%; moderate, >2%.^7^

### Assessment of isothiocyanates in modified Direct Peptide Reactivity Assays (DPRA)

In the DPRA procedure, developed by Gerberick *et al*.^3^, ACN or DMSO is used to dissolve the haptens. Unfortunately, 6-TRITC did not dissolve in ACN and when DMSO was used complete dimerization of the cysteine peptide was observed in the control samples after 24 h. Therefore, MeOH was used to dissolve the isothiocyanates; the total MeOH content in the final reaction mixture was 25%. The original DPRA method developed by Gerberick *et al*. uses phosphate buffer pH 7.5 and ammonium acetate buffer pH 10.2 for the cysteine peptide and lysine peptide, respectively. However, 6-TRITC (and FITC) proved to be unstable in the ammonium acetate buffer and was completely degraded after only 2 h. As 6-TRITC degraded in ammonium acetate buffer, the same reaction conditions were used for both peptides, i.e. 25% MeOH in phosphate buffer pH 7.5. Stock solutions of cysteine peptide (Ac-RFAA**C**AA-COOH), lysine peptide (Ac-RFAA**K**AA-COOH), and PHCKRM were prepared in sodium phosphate buffer (100 mM, pH 7.5), while isothiocyanates were diluted in MeOH. The reactions were performed in HPLC vials after adding the solutions as specified below. The vials were capped, vortexed, and kept in the autosampler at room temperature (in the dark) for the entire analysis time. Samples were taken every 40 min for 24 h and analyzed with HPLC/ESI-MS. Standard curves of the peptides (Figures S10–S11) were used to measure peptide depletion and mass data was used to confirm that the depletion was due to isothiocyanate-peptide adduct formation.

#### Reactions of isothiocyanates with cysteine or lysine peptides (10:1)

For each reactivity experiment, 75 μL of peptide solution (2.0 mM), 75 μL of isothiocyanate solution (20 mM) and 150 μL of phosphate buffer were added to the vials.

#### Reactions of isothiocyanates with cysteine or lysine peptides (1:1)

For each experiment, an aliquot of 75 μL of peptide solution (2.0 mM), 75 μL of isothiocyanate solution (2.0 mM) and 150 μL of phosphate buffer were added to the vials.

#### Reactions of isothiocyanates in presence of both cysteine and lysine peptides (1:1:1)

For each experiment, 75 μL of isothiocyanate solution (2.0 mM), 75 μL of each peptide solution (2.0 mM) and 75 μL of buffer were added to the vials.

#### Reactions of isothiocyanates with the hexapeptide PHCKRM (10:1)

For each experiment, 75 μL of isothiocyanate solutions (20 mM), 150 μL of peptide solution (1.0 mM), and 75 μL of phosphate buffer were added to the vials.

#### Reactions of isothiocyanates with the hexapeptide PHCKRM (1:1)

For each experiment, 75 μL of isothiocyanate solution (2.0 mM), 150 μL of peptide solution (1.0 mM), and 75 μL of phosphate buffer were added to the vials. The samples were thereafter handled, analyzed and quantified as described in the general procedure, except that the HPLC gradient was changed to 20% B to 60% B over 30 min.

### Computational Techniques

Calculations were performed on a mixture of the C3SE cluster (SNIC facility located at Chalmers University of Technology, Gothenburg) and standalone workstations running CentOS 6.6. All DFT calculations were carried out at the B3LYP-D3/6-31+G**^23^,^24^,^25^,^26^,^27^,^28^,^29^,^30^,^31^,^32^,^33^,^34^,^35^ level of theory in Jaguar (Version 8.0, Schrodinger LLC, New York, NY, USA, 2013). Implicit solvation was used (PBF model). Transition states, using structures prepared in Macromodel (Version 9.9, Schrodinger LLC, New York, NY, USA, 2013) and Maestrop (Version 9.4, Schrodinger LLC, New York, NY, USA, 2013), were deduced initially using the LST method, with variation of the initial guess where necessary. MeNH_2_ was used as a model for amine groups in the peptide, and MeSH for cysteine sulfur units, to reduce the computational load.

## Additional Information

**How to cite this article**: Karlsson, I. *et al*. Peptide Reactivity of Isothiocyanates – Implications for Skin Allergy. *Sci. Rep.* **6**, 21203; doi: 10.1038/srep21203 (2016).

## Supplementary Material

Supplementary Information

## Figures and Tables

**Figure 1:**
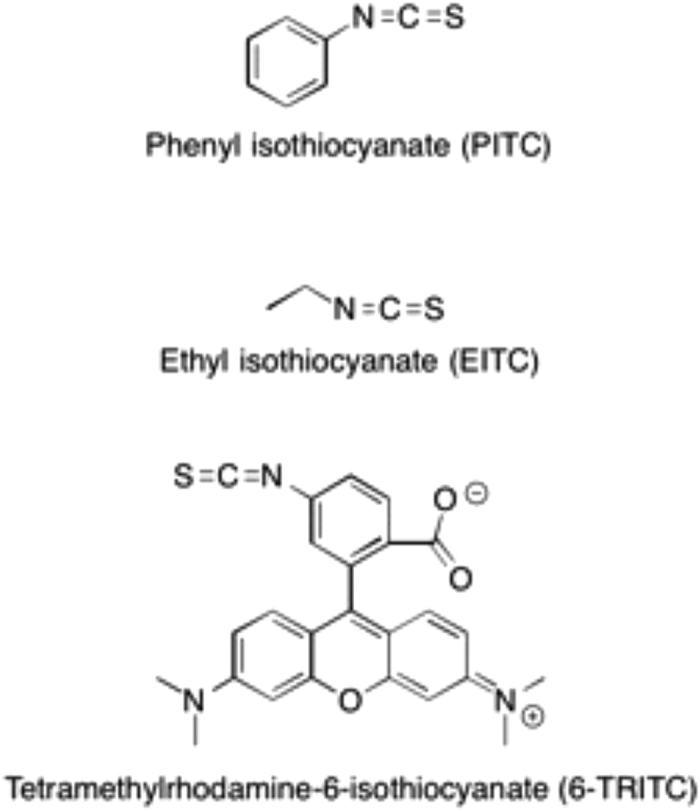
Compounds investigated in this study.

**Table 1:** Reactivity of isothiocyanates toward cysteine and lysine peptides measured after 24 h with results expressed as percent depletion of peptide.

	Cysteine peptide/test compound (1:10)	Lysine peptide/test compound (1:10)	Average peptide depletion
6-TRITC	100	31	66
PITC	99	13	56
EITC	100	7.8	54

**Table 2:** Peptide adducts detected in the reactivity experiments with isothiocyanates/PHCKRM (10:1).

	Time	Mono-adduct Cys	Mono-adduct Pro	Di-adduct	Tri-adduct
6-TRITC	2 min	Minor	Minor	Major	Minor
	12 h	Trace	Trace	Minor	Major
	24 h	Trace	Trace	Minor	Major
PITC*	2 min	Major	Minor	Minor	Trace
	12 h	Trace	Minor	Major	Minor
	24 h	Trace	Minor	Major	Minor
EITC*	2 min	Major	Minor	Trace	Not detected
	12 h	Major	Minor	Minor	Not detected
	24 h	Major	Minor	Minor	Not detected

^*^The Cys and Pro mono-adducts co-elute; therefore, the fragmentation pattern (Tables S3 and S4) was used to determine which adduct that was the major one.

**Table 3:** Peptide adducts detected in the reactivity experiments with isothiocyanates/PHCKRM (1:1).

	Time	Mono-adduct Cys	Mono-adduct Pro	Di-adduct	Tri-adduct	Hapten-pep# dimer
6-TRITC	2 min	**Major**	Trace	Minor	Not detected	Not detected
	12 h	Minor	Minor	**Major**	Trace	Trace
	24 h	Trace	Minor	**Major**	Minor	Minor
PITC*	2 min	**Major**	Minor	Not detected	Not detected	Not detected
	12 h	Minor	**Major**	Minor	Not detected	Trace
	24 h	Trace	**Major**	Minor	Not detected	Minor
EITC*	2 min	**Major**	Minor	Not detected	Not detected	Not detected
	12 h	**Major**	Minor	Trace	Not detected	Not detected
	24 h	**Major**	Minor	Trace	Not detected	Not detected

^#^For 6-TRITC the adduct is (hapten-PHCKRM)_2_ and for PITC the adduct is hapten-(PHCKRM)_2_

^*^The Cys and Pro mono-adducts co-elute; therefore, the fragmentation pattern (Tables S6 and S7) was used to determine which adduct that was the major one.
