# Supplementary material for: Peptide Reactivity of Isothiocyanates – Implications for Skin Allergy
Source: Sci Rep. 2016 Feb 17;6:21203. doi: 10.1038/srep21203 (PMC4756319; doi:10.1038/srep21203)
Supplement: Supplementary Information [file srep21203-s1.pdf]

SUPPORTING INFORMATION FOR:

PEPTIDE REACTIVITY OF ISOTHIOCYANATES – IMPLICATIONS FOR SKIN ALLERGY

Isabella Karlsson,[†] *Kristin Samuelsson,*[‡] *David J. Ponting,* [‡] *Margareta Törnqvist,* [†] *Leopold L. Ilag,*[†] *and Ulrika Nilsson*[†,*]

[†]Department of Environmental Science and Analytical Chemistry, Stockholm University, SE-106 91 Stockholm, Sweden

[‡] Dermatochemistry and Skin Allergy, Department of Chemistry and Molecular Biology, University of Gothenburg, SE-412 96 Gothenburg, Sweden

TABLE OF CONTENTS

Local Lymph Node Assay (LLNA) ... 3
- Table S1. LLNA responses for TRITC ... 3

DPRA of Isothiocyanates toward Cysteine and Lysine peptides ... 4
- Figure S1: Representative chromatograms DPRA ... 5

Stability of Isothiocyanates ... 6
- Figure S2: Stability of isothiocyanates ... 6

DPRA of FITC toward Lysine Peptide (10:1) ... 7
- Figure S3. Lysine peptide and FITC (1:10) ... 7

DPRA of Isothiocyanates toward PHCKRM ... 8
- PHCKRM and 6-TRITC (1:10) ... 8
  - Figure S4. PHCKRM and 6-TRITC (1:10) ... 8
  - Table S2. Adducts and fragment ions from the reaction of PHCKRM and 6-TRITC (1:10) ... 9
- PHCKRM and PITC (1:10) ... 10
  - Figure S5. PHCKRM and PITC (1:10) ... 10
  - Table S3. Adducts and fragment ions from the reaction of PHCKRM and PITC (1:10) ... 11
- PHCKRM and EITC (1:10) ... 12
  - Figure S6. PHCKRM and EITC (1:10) ... 12
  - Table S4. Adducts and fragment ions from the reaction of PHCKRM and EITC (1:10) ... 13
- PHCKRM and 6-TRITC (1:1) ... 14
  - Figure S7. PHCKRM and 6-TRITC (1:1) ... 15
  - Table S5. Adducts and fragment ions from the reaction of PHCKRM and 6-TRITC (1:1) ... 16
- PHCKRM and PITC (1:1) ... 17
  - Figure S8. PHCKRM and PITC (1:1) ... 17
  - Table S6. Adducts and fragment ions from the reaction of PHCKRM and PITC (1:1) ... 18
- PHCKRM and EITC (1:1) ... 19
  - Figure S9. PHCKRM and EITC (1:1) ... 19
  - Table S7. Adducts and fragment ions from the reaction of PHCKRM and EITC (1:1) ... 20

Standard Curve for Lysine Peptide ... 21
- Table S8: Standard curve for lysine peptide used in the experiments with 6-TRITC, PITC, and EITC ... 21
- Figure S10: Standard curve for lysine peptide used in the experiments with 6-TRITC, PITC, and EITC ... 21
- Table S9: Standard curve for lysine peptide used in the experiments with FITC ... 22
- Figure S11: Standard curve for lysine peptide used in the experiments with FITC ... 22

Local Lymph Node Assay (LLNA)

The sensitizing potency of 6-TRITC was investigated using the murine LLNA, approved by the local ethics committee. 6-TRITC was tested at five different concentrations using mice in groups of three (table S1). Groups of female CBA/CA mice received 25 µL of a solution of test compound, dissolved in the vehicle acetone/dibutyl phtalate (1:1 v/v), on the dorsum of the ears daily for three consecutive days. Control animals were treated in the same way with vehicle alone. All mice were injected intravenously 5 days after the first treatment, with 250 µL of PBS (pH 7.4, 137 mM NaCl, 2.7 mM KCl, 10 mM phosphate buffer solution) containing 20 µCi of [^{3}H]-thymidine. Five hours later, the draining lymph nodes were excised and pooled for each group, and a single cell suspension of lymph node cells was prepared. The thymidine incorporation was measured by β-scintillation counting. Results are expressed as the mean dpm/lymph node for each experimental group and as stimulation index (SI). The stimulation index is defined as the ratio between dpm/lymph node for the test group and the control group. Compounds that at one or more concentrations cause an SI greater than 3 are considered to be positive in the LLNA. EC3 values (the estimated concentration required to induce an SI of 3) are calculated by linear interpolation. The sensitizing potency of test compounds is classified according to the following: extreme, ≤0.2%; strong, >0.2% to ≤2%; moderate, >2%.

Table S1. LLNA responses for TRITC

	6-TRITC			
	Concentration		DPM/	SI[1]
	%	mM	node	
	A:DBP[2]		1311	
	0.00010	0.0023	969	0.74
	0.0010	0.023	902	0.69
	0.010	0.23	2688	2.1
	0.10	2.3	6164	4.7
	1.0	23	16700	13
EC3[3]	**0.040**	**0.92**		

6-TRITC, 6-tetramethylrhodamine isothiocyanate

[1] An increase in thymidine incorporation relative to vehicle-treated controls was derived for each experimental group and recorded as a stimulation index (SI).

[2] Acetone:dibutyl phthalate (A:DBP) related as 1:1.

[3] EC3 is the estimated concentration required to induce and SI of 3 calculated using linear interpolation.

DPRA of Isothiocyanates toward Cysteine and Lysine peptides

Representative chromatograms from the DPRA experiments using isothiocyanates and cysteine (Ac-RFAA**C**AA-COOH) and lysine (Ac-RFAA**K**AA-COOH) peptides respectively are shown in figure S1. The experiments were performed as described in the manuscript.

a.

CYS-PEPTIDE (0.5 mM) PITC (5 mM) 2 min

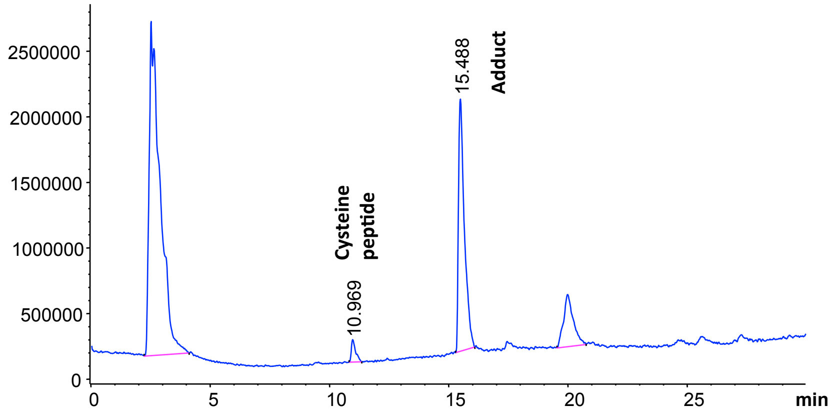

b.

CYS-PEPTIDE (0.5 mM) EITC (5 mM) 2 min

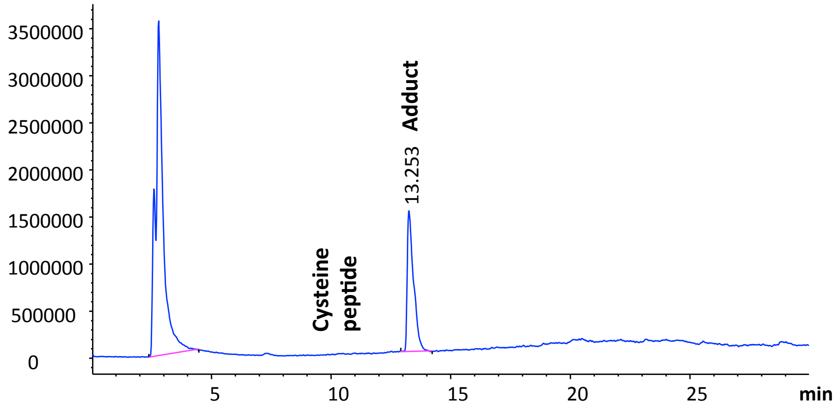

c.

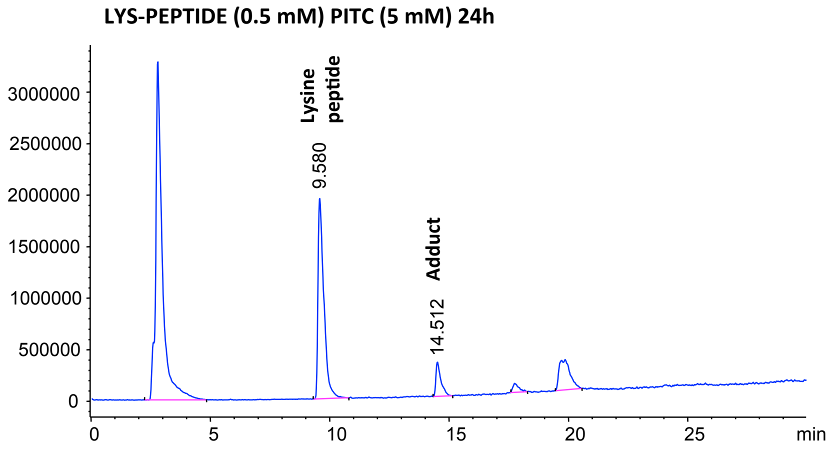

d.

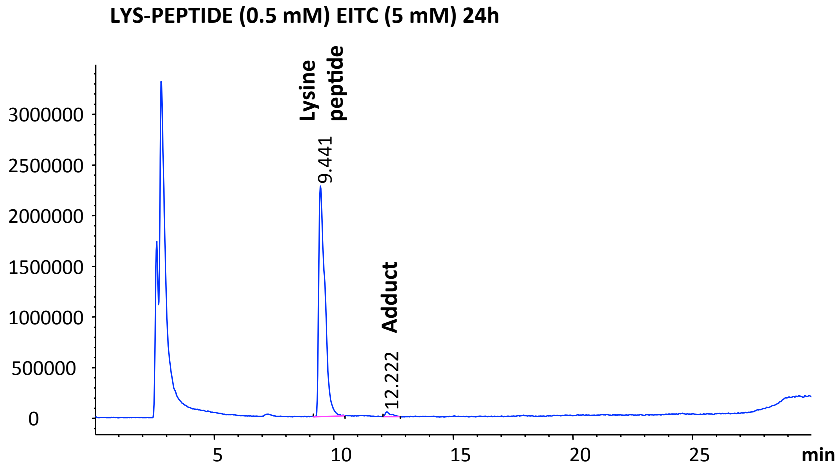

Figure S1: Representative chromatograms DPRA

ESI-MS (TIC) spectra showing: (**a**) cysteine peptide and adduct formed from PITC and the cysteine peptide after 2 min of reaction time; (**b**) cysteine peptide and adduct formed from EITC and the cysteine peptide after 2 min of reaction time; (**c**) lysine peptide and adduct formed from PITC and the lysine peptide after 24 h of reaction time; and (**d**) lysine peptide and adduct formed from EITC and the lysine peptide after 24 h of reaction time.

Stability of Isothiocyanates

The stability of the isothiocyanates during the reaction conditions were evaluated by preparing 20 mM stock solutions in MeOH, which were diluted with phosphate buffer pH 7.5 to 5 mM. The samples were analyzed every 80 min for 24 h, see figure S2.

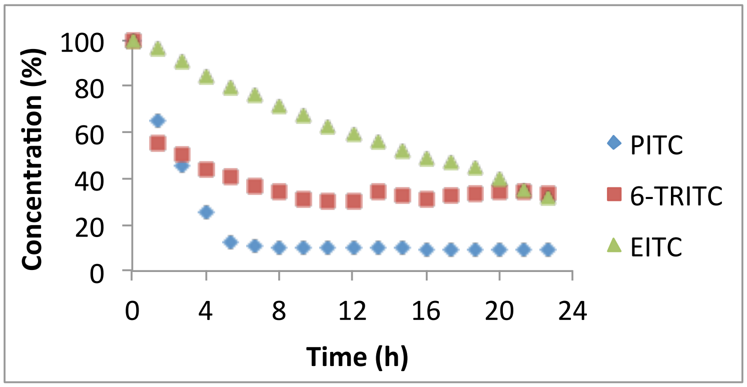

Figure S2: Stability of isothiocyanates

Degradation of the isothiocyanates in the reaction media, MeOH/phosphate buffer pH 7.5 (1:3), over 24 h: *blue diamond,* Phenyl isothiocyanate (PITC); *green triangle,* Ethyl isothiocyanate (EITC); *red square,* 6-Tetramethylrhodamine isothiocyanate (6-TRITC).

DPRA of FITC toward Lysine Peptide (10:1)

The reactivity of FITC toward the lysine peptide was assessed using the same conditions as for 6-TRITC, PITC, and EITC, i.e. 5 mM of FITC and 0.5 mM of lysine peptide (Ac-RFAAKAA-COOH) in MeOH/phosphate buffer pH 7.5 (1:3).

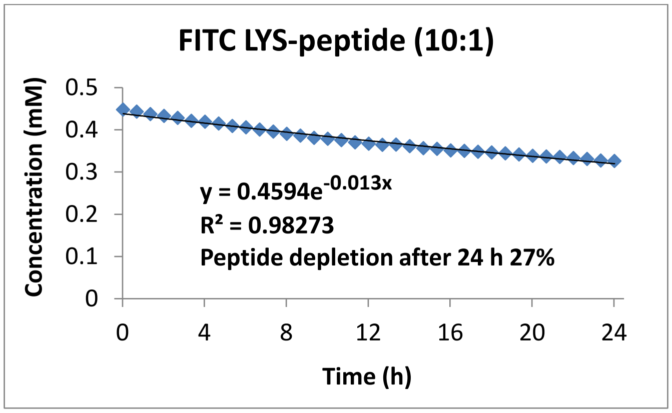

Figure S3. Lysine peptide and FITC (1:10)

Concentration of the lysine peptide (Ac-RFAA**K**AA-COOH, 0.5 mM) over time in the DPRA with FITC (5 mM).

DPRA of Isothiocyanates toward PHCKRM

DPRA experiments of the isothiocyanates 6-TRITC, PITC, and EITC toward the peptide PHCKRM was performed as described in the manuscript. Chromatograms and the detected parent and fragment ions are shown below.

PHCKRM and 6-TRITC (1:10)

a.

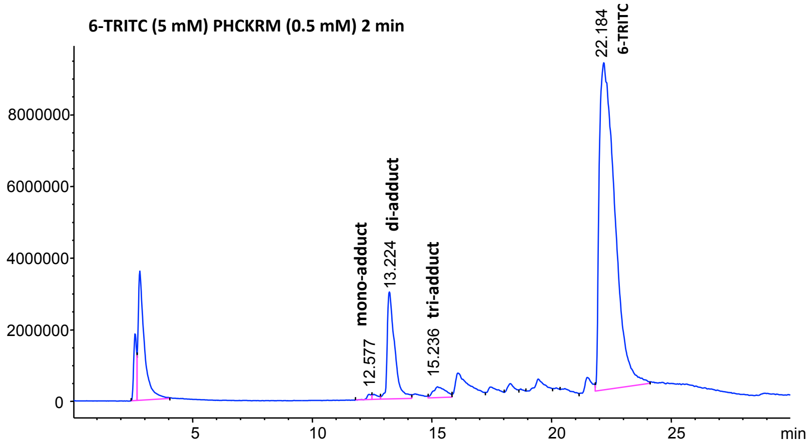

b.

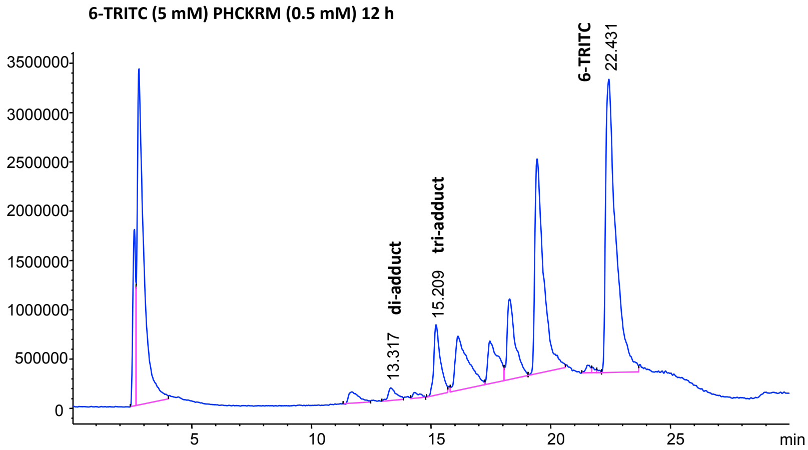

Figure S4. PHCKRM and 6-TRITC (1:10)

The figure shows the ESI-MS TIC chromatogram for reactivity experiment with 6-TRITC (5 mM) and the hexapeptide PHCKRM (0.5 mM) after (**a**) 2 min and (**b**) 12 h.

Table S2. Adducts and fragment ions from the reaction of PHCKRM and 6-TRITC (1:10)

	m/z	Mono-add. (Cys) 2 min	Mono-add. (Pro) 2 min	Di-add. 2 min	Di-add. 12 h	Tri-add. 12 h	Tri-add. 24 h
**$[PHCKRM+TRITC+H]^{+}$**	1214.3	√	√	√	√	√	√
**$[PHCKRM+TRITC+2H]^{2+}$**	607.8	√	√	√	√	√	√
**$[PHCKRM+TRITC+3H]^{3+}$**	405.7	√	√				
**$[PHCKRM+2TRITC+2H]^{2+}$**	829.4			√	√	√	√
**$[PHCKRM+2TRITC+3H]^{3+}$**	553.4			√	√	√	√
**$[PHCKRM+2TRITC+4H]^{4+}$**	415.4			√	√		
**$[PHCKRM+3TRITC+2H]^{2+}$**	1050.7					√	√
**$[PHCKRM+3TRITC+3H]^{3+}$**	701.2					√	√
**$[PHCKRM+3TRITC+4H]^{4+}$**	526.2					√	√
**[a]$[y5+TRITC+2H]^{2+}$**	559.1	√					
**[b]$[b1+TRITC+2H]^{2+}$**	271.1		√				
**y5**	674.3		√				
**y4**	537.3		√				
**$[PHCKRM+H]^{+}$**	771.3	√	√	√	√	√	√
**$[6\text{-}TRITC+H]^{+}$**	444.2	√	√	√	√	√	√

[a] TRITC is on either H, C, K, R, or, M

[b] TRITC is on P

PHCKRM and PITC (1:10)

a.

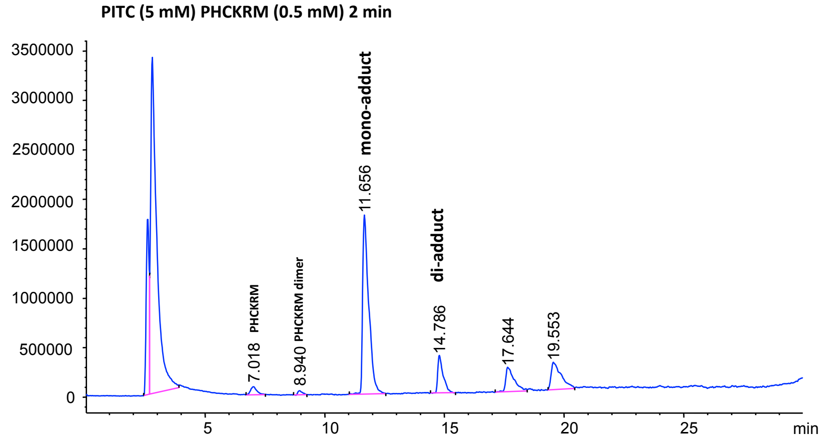

b.

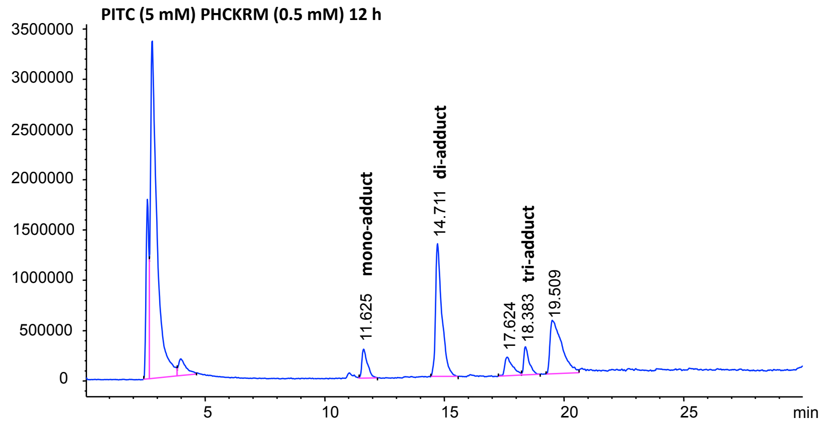

Figure S5. PHCKRM and PITC (1:10)

The figure shows the ESI-MS TIC chromatogram for reactivity experiment with PITC (5 mM) and the hexapeptide PHCKRM (0.5 mM) after (**a**) 2 min and (**b**) 12 h.

Table S3. Adducts and fragment ions from the reaction of PHCKRM and PITC (1:10)

	m/z	Mono-add. 2 min	Mono-add. 12 h	Mono-add. 24 h	Di-add. 2 min	Di-add. 12 h	Di-add. 24 h	Tri-add. 12 h	Tri-add. 24 h
**[PHCKRM+PITC+H]$^{+}$**	906.4	√	√	√	√	√	√		
**[PHCKRM+PITC+2H]$^{2+}$**	453.8	√	√	√	√	√	√		
**[PHCKRM+PITC+3H]$^{3+}$**	303.0	√	√	√					
**[PHCKRM+2PITC+H]$^{+}$**	1041.3				√	√	√	√	√
**[PHCKRM+2PITC+2H]$^{2+}$**	521.3				√	√	√	√	√
**[PHCKRM+2PITC+3H]$^{3+}$**	347.9				√	√	√		
**[PHCKRM+3PITC+H]$^{+}$**	1176.0							√	√
**[PHCKRM+3PITC+2H]$^{2+}$**	588.8							√	√
**[PHCKRM+3PITC+3H]$^{3+}$**	393.0							√	√
**[a][y4+PITC+H]$^{+}$**	672.3	√			√	√			
**[b][y5+PITC+H]$^{+}$**	809.3	√			√	√	√		
**[b][y5+PITC+2H]$^{2+}$**	405.3	√			√	√	√		
**[c][y5+2PITC+H]$^{+}$**	944.2							√	√
**[c][y5+2PITC+2H]$^{2+}$**	472.8							√	√
**[d][b1+PITC+H]$^{+}$**	233.1		√	√	√	√	√	√	√
**[e][b3+2PITC+H]$^{+}$**	607.7							√	√
**y5**	674.3	√	√	√					
**y4**	537.3	√	√	√					
**[PHCKRM+H]$^{+}$**	771.3	√	√	√	√	√	√	√	√
**[PITC+H]$^{+}$**	136.2								

[a] PITC is on either C, K, R, or, M

[b] PITC is on either H, C, K, R, or, M

[c] Two PITC on H, C, K, R, and/or, M

[d] PITC is on P

[e] Two PITC on P, H, and/or C

PHCKRM and EITC (1:10)

a.

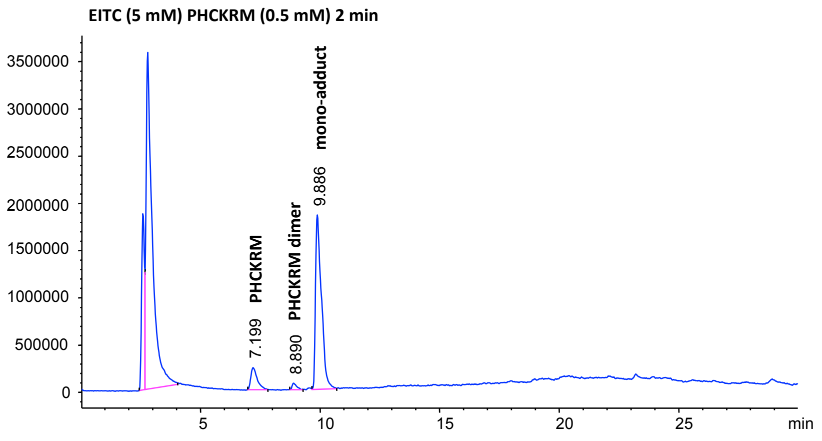

b.

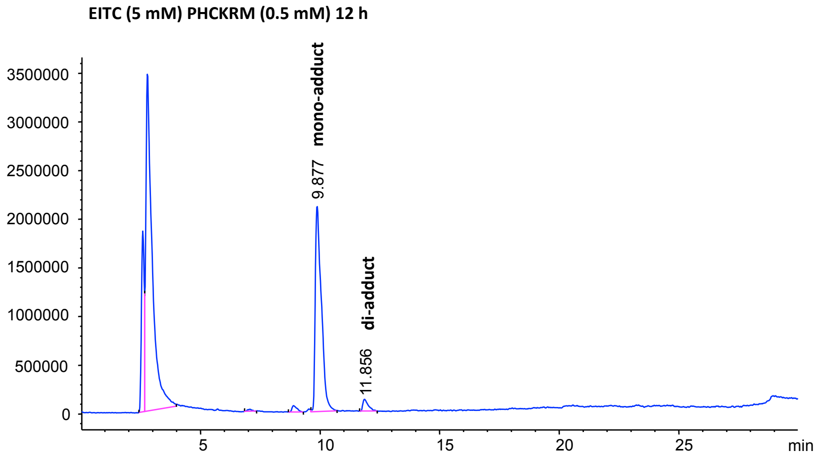

Figure S6. PHCKRM and EITC (1:10)

The figure shows the ESI-MS TIC chromatogram for reactivity experiment with EITC (5 mM) and the hexapeptide PHCKRM (0.5 mM) after (**a**) 2 min and (**b**) 12 h.

Table S4. Adducts and fragment ions from the reaction of PHCKRM and EITC (1:10)

	m/z	Mono-add. 2 min	Mono-add. 12 h	Mono-add. 24 h	Di-add. 12 h	Di-add. 24 h
**$[PHCKRM+EITC+H]^+$**	858.4	√	√	√		
**$[PHCKRM+EITC+2H]^{2+}$**	429.8	√	√	√	√	√
**$[PHCKRM+EITC+3H]^{3+}$**	286.9	√	√	√		
**$[PHCKRM+2EITC+H]^+$**	945.4				√	√
**$[PHCKRM+2EITC+2H]^{2+}$**	473.3				√	√
**$[PHCKRM+2EITC+3H]^{3+}$**	316.0				√	√
**[a]$[y4+EITC+H]^+$**	624.3	√	√	√	√	
**[b]$[y5+EITC+H]^+$**	761.3	√	√	√	√	√
**[b]$[y5+EITC+2H]^{2+}$**	381.2	√	√	√	√	√
**[c]$[b1+EITC+H]^+$**	185.2				√	√
**y5**	674.3	√	√	√	√	
**y4**	537.3	√	√	√	√	
**$[PHCKRM+H]^+$**	771.3	√	√	√	√	√
**$[EITC +H]^+$**	88.2					

[a] EITC is on either C, K, R, or, M

[b] EITC is on either H, C, K, R, or, M

[c] EITC is on P

PHCKRM and 6-TRITC (1:1)

a.

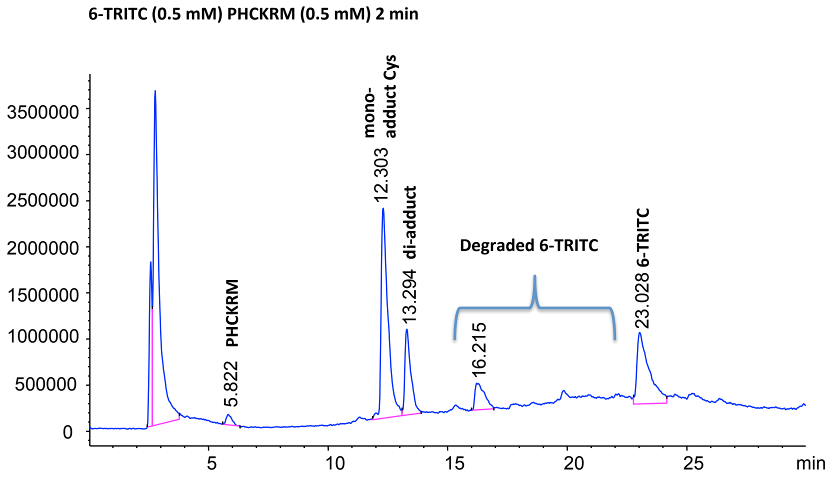

b.

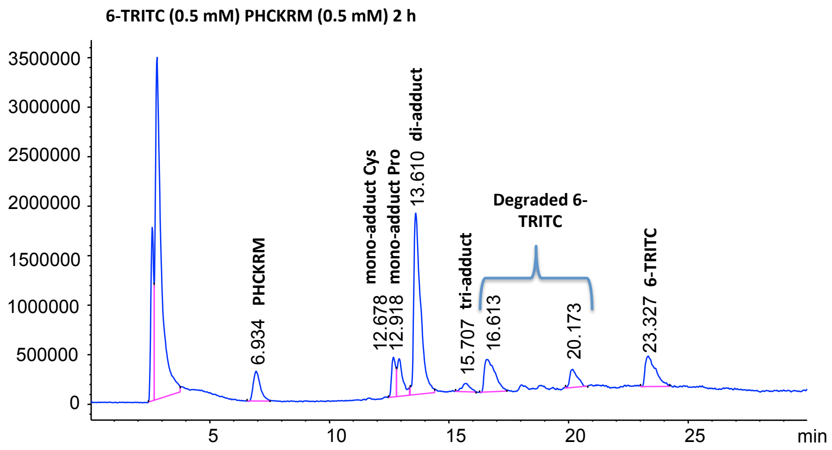

c.

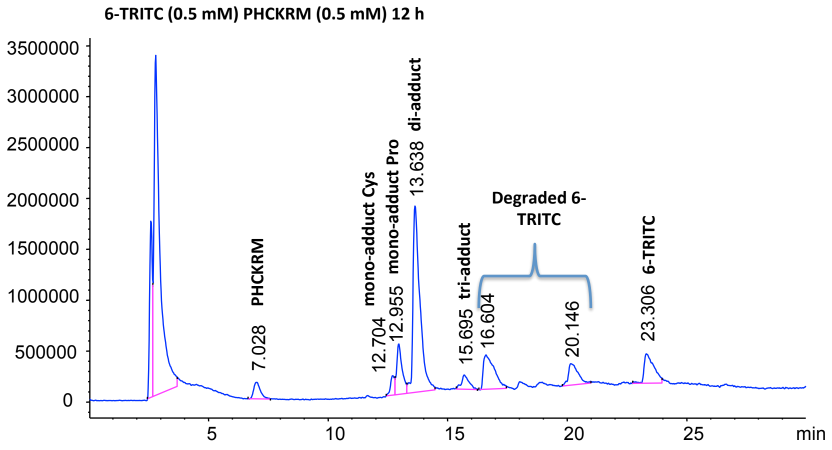

d.

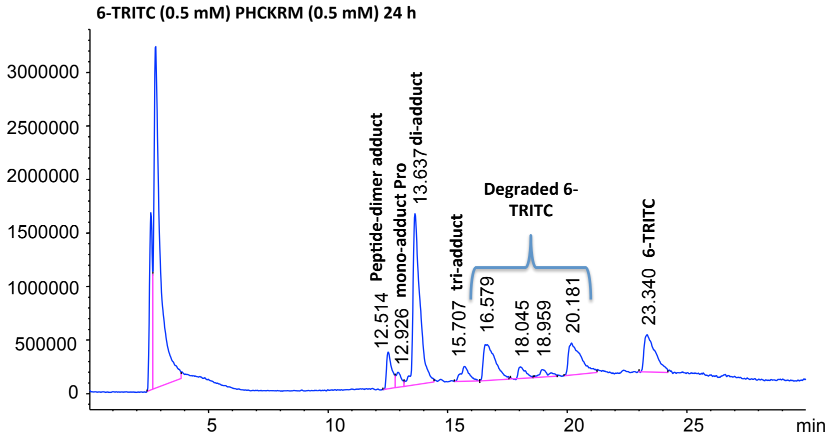

Figure S7. PHCKRM and 6-TRITC (1:1)

The figure shows the ESI-MS TIC chromatogram for reactivity experiment with 6-TRITC (0.5 mM) and the hexapeptide PHCKRM (0.5 mM) after (**a**) 2 min, (**b**) 2 h, (**c**) 12 h, and (**d**) 24 h.

Table S5. Adducts and fragment ions from the reaction of PHCKRM and 6-TRITC (1:1)

	m/z	Mono-add. (Cys) 2 min	Mono-add. (Pro) 2 h	Di-add. 2 min	Di-add. 12 h	Tri-add. 24 h	(TRITC-pep) dimer 24 h
**$[PHCKRM+TRITC+H]^{+}$**	1214.3	√	√	√	√	√	
**$[PHCKRM+TRITC+2H]^{2+}$**	607.8	√	√	√	√	√	
**$[PHCKRM+TRITC+3H]^{3+}$**	405.7	√	√				
**$[PHCKRM+2TRITC+2H]^{2+}$**	829.4			√	√	√	
**$[PHCKRM+2TRITC+3H]^{3+}$**	553.4			√	√	√	
**$[PHCKRM+2TRITC+4H]^{4+}$**	415.4			√	√		
**[a]$[y4+TRITC+H]^{+}$**	1050.7	√					
**[a]$[y4+TRITC+2H]^{2+}$**	701.2	√					
**[b]$[y5+TRITC+2H]^{2+}$**	526.2	√				√	
**[c]$[b1+TRITC+H]^{+}$**	559.1		√				
**[c]$[b1+TRITC+2H]^{2+}$**	271.1		√		√	√	
**y5**	674.3		√				√
**y4**	537.3		√				√
**$[(PHCKRM+TRITC)_2+2H]^{2+}$**	537.3						√
**$[(PHCKRM+TRITC)_2+3H]^{3+}$**	537.3						√
**$[(PHCKRM+TRITC)_2+4H]^{4+}$**	537.3						√
**$[(PHCKRM+TRITC)_2-TRITC+2H]^{2+}$**	537.3						√
**$[(PHCKRM+TRITC)_2-TRITC+3H]^{3+}$**	537.3						√
**$[PHCKRM+H]^{+}$**	771.3	√	√	√	√	√	√
**$[6-TRITC+H]^{+}$**	444.2	√	√	√	√	√	√

[a] TRITC is on either C, K, R, or, M

[b] TRITC is on either H, C, K, R, or, M

[c] TRITC is on P

PHCKRM and PITC (1:1)

a.

PITC (0.5 mM) PHCKRM (0.5 mM) 2 min

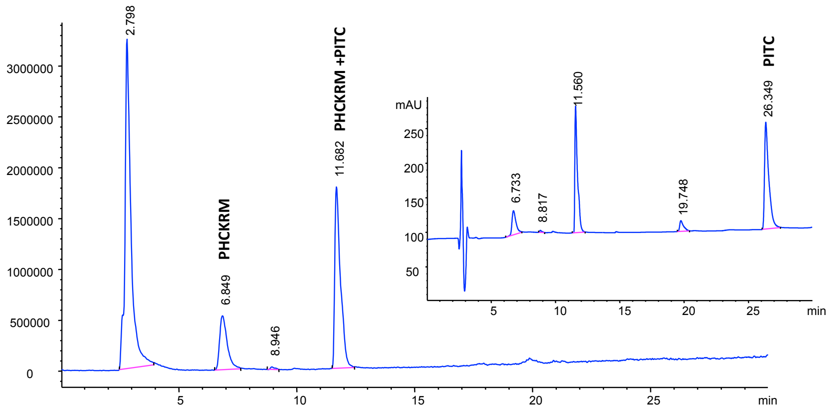

b.

PITC (0.5 mM) PHCKRM (0.5 mM) 12 h

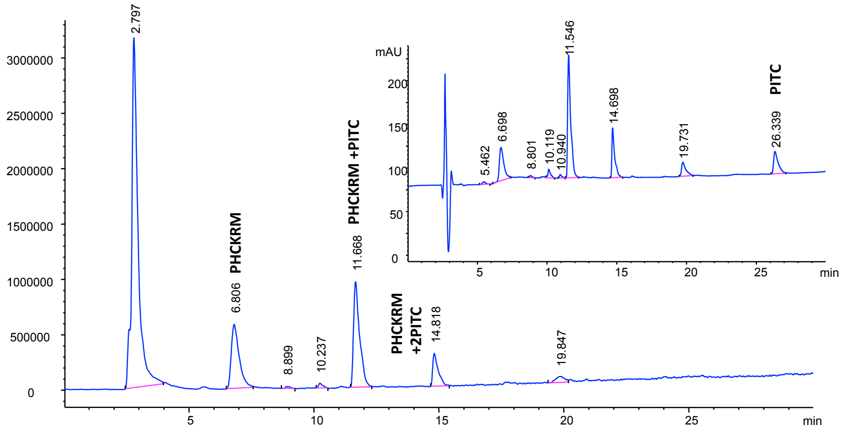

c.

PITC (0.5 mM) PHCKRM (0.5 mM) 24 h

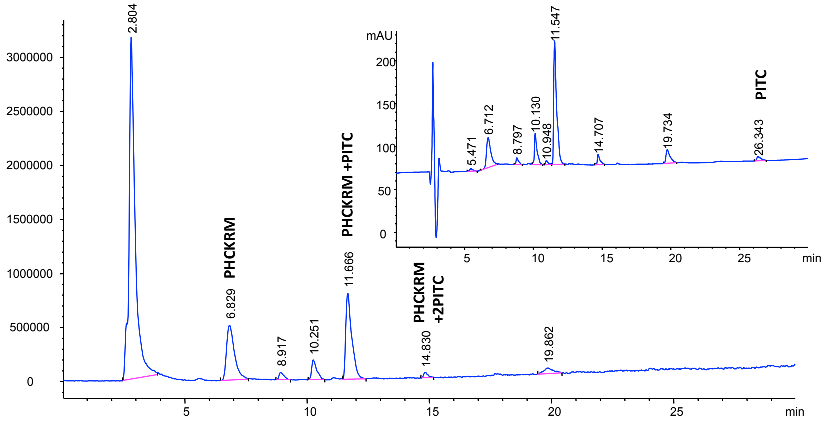

Figure S8. PHCKRM and PITC (1:1)

The figure shows the ESI-MS TIC chromatogram for reactivity experiment with PITC (0.5 mM) and the hexapeptide PHCKRM (0.5 mM) after: (**a**) 2 min, (**b**) 12 h, and (**c**) 24 h.

Table S6. Adducts and fragment ions from the reaction of PHCKRM and PITC (1:1)

	m/z	Mono-add. 2 min	Mono-add. 12 h	Mono-add. 24 h	Di-add. 12 h	Di-add. 24 h	PITC-(pep-dimer) 12 h	PITC-(pep-dimer) 24 h
**$[PHCKRM+PITC+H]^+$**	906.4	√	√	√	√	√		
**$[PHCKRM+PITC+2H]^{2+}$**	453.8	√	√	√	√	√		
**$[PHCKRM+PITC+3H]^{3+}$**	303.0	√	√	√				
**$[PHCKRM+2PITC+H]^+$**	1041.3				√	√		
**$[PHCKRM+2PITC+2H]^{2+}$**	521.3				√	√		
**$[PHCKRM+2PITC+3H]^{3+}$**	347.9				√	√		
**[a]$[y4+PITC+H]^+$**	672.3	√			√			
**[b]$[y5+PITC+H]^+$**	809.3	√			√	√		
**[b]$[y5+PITC+2H]^{2+}$**	405.3	√	trace		√	√		
**[c]$[b1+PITC+H]^+$**	233.1		√	√	√	√		
**y5**	674.3	√	√	√	√	√		
**y4**	537.3	√	√	√				
**$[(PHCKRM)_2PITC+H+Na]^{2+}$**	849.3						√	√
**$[(PHCKRM)_2PITC+2H]^{2+}$**	837.9						√	√
**$[PITC(PHCKRM)_2+3H]^{3+}$**	559.1						√	√
**$[PITC(PHCKRM)_2+4H]^{4+}$**	419.7						√	√
**$[PHCKRM+y5+2H]^{2+}$**	721.9						√	√
**$[PHCKRM+H]^+$**	771.3	√	√	√	√	√	√	√
**$[PITC+H]^+$**	136.2							

[a] PITC is on either C, K, R, or, M

[b] PITC is on either H, C, K, R, or, M

[c] PITC is on P

PHCKRM and EITC (1:1)

a.

EITC (0.5 mM) PHCKRM (0.5 mM) 2 min

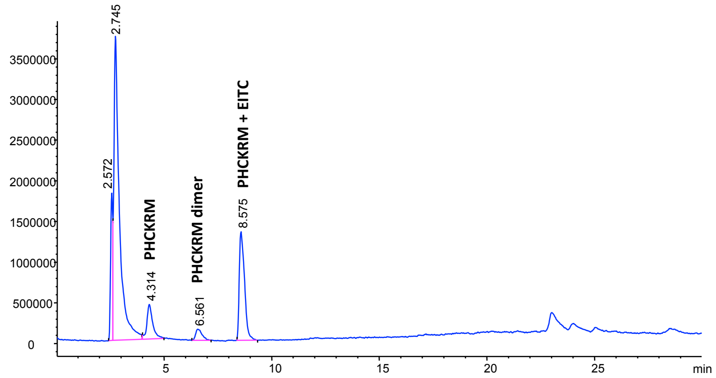

b.

EITC (0.5 mM) PHCKRM (0.5 mM) 12 h

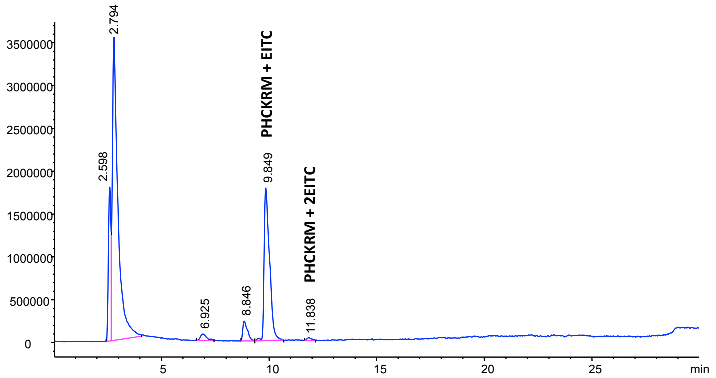

c.

EITC (0.5 mM) PHCKRM (0.5 mM) 1442 min

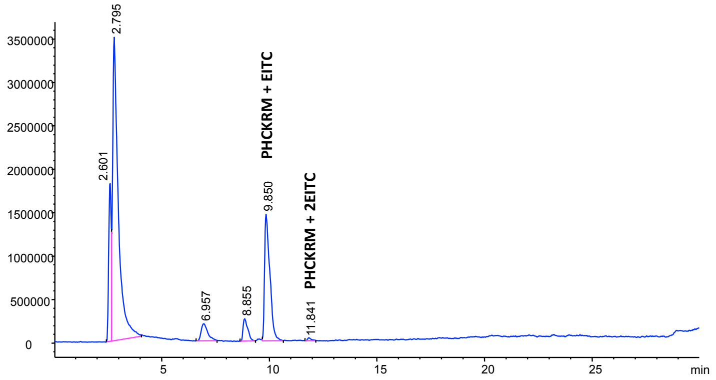

Figure S9. PHCKRM and EITC (1:1)

The figure shows the ESI-MS TIC chromatogram for reactivity experiment with EITC (0.5 mM) and the hexapeptide PHCKRM (0.5 mM) after: (**a**) 2 min, (**b**) 12 h, and (**c**) 24 h.

Table S7. Adducts and fragment ions from the reaction of PHCKRM and EITC (1:1)

	m/z	Mono-add. 2 min	Mono-add. 12 h	Mono-add. 24 h	Di-add. 12 h	Di-add. 24 h
**[PHCKRM+EITC+Na]$^{+}$**	880.3	√	√	√		
**[PHCKRM+EITC+H]$^{+}$**	858.4	√	√	√		
**[PHCKRM+EITC+2H]$^{2+}$**	429.8	√	√	√	√	√
**[PHCKRM+EITC+3H]$^{3+}$**	286.9	√	√	√		
**[PHCKRM+EITC+Na+H]$^{2+}$**	440.8	√	√	√		
**[PHCKRM+2EITC+H]$^{+}$**	945.4				√	√
**[PHCKRM+2EITC+2H]$^{2+}$**	473.3				√	√
**[PHCKRM+2EITC+3H]$^{3+}$**	316.0				√	√
**[PHCKRM+2EITC+Na+H]$^{2+}$**	484.3				√	√
**[a][y4+EITC+H]$^{+}$**	624.3	√	√	√		
**[b][y5+EITC+H]$^{+}$**	761.3	√	√	√	√	√
**[b][y5+EITC+2H]$^{2+}$**	381.2	√	√	√	√	√
**[c][b1+EITC+H]$^{+}$**	185.2				√	√
**y5**	674.3	√	√	√		
**y4**	537.3	√	√	√		
**[PHCKRM+H]$^{+}$**	771.3	√	√	√	√	√
**[EITC +H]$^{+}$**	88.2					

[a] EITC is on either C, K, R, or, M

[b] EITC is on either H, C, K, R, or, M

[c] EITC is on P

Standard Curve for Lysine Peptide

Measuring the peak area in the HPLC/UV chromatogram recorded at 220 nm produced a standard curve for the lysine peptide. A 2.0 mM stock solution of lysine peptide in MeOH/phosphate buffer pH 7.5 (1:3) was made. The stock solution was then diluted with MeOH/phosphate buffer pH 7.5 (1:3) to concentrations ranging from 0.75 mM to 0.0156 mM (table S8).

Table S8: Standard curve for lysine peptide used in the experiments with 6-TRITC, PITC, and EITC

Concentration (mM)	Area peptide (220 nm)
0.0156	61.5173
0.0156	58.5851
0.0313	97.4073
0.0313	101.337
0.0625	176.748
0.0625	175.32
0.125	386.23
0.125	390.212
0.25	757.076
0.25	764.567
0.5	1547.28
0.5	1554.22
0.75	2401.08
0.75	2414.77

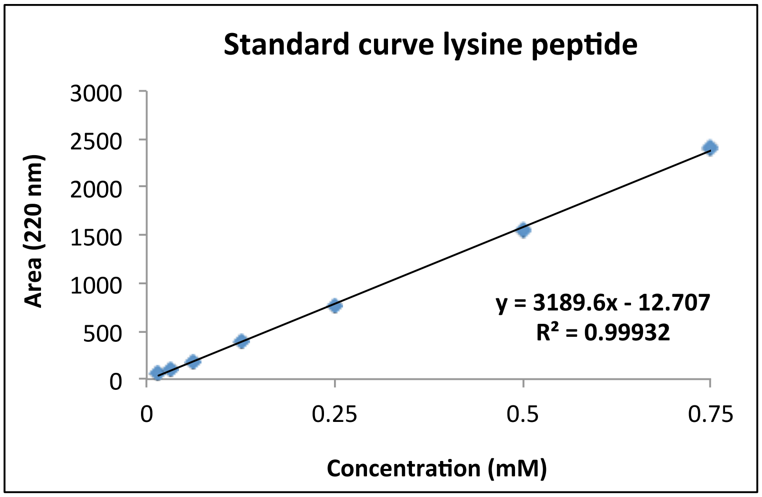

Figure S10: Standard curve for lysine peptide used in the experiments with 6-TRITC, PITC, and EITC

The peak areas of the lysine peptide at 220 nm are plotted against the concentrations and the obtained *k, m* and R^2 values are shown.

Table S9: Standard curve for lysine peptide used in the experiments with FITC

Concentration (mM)	Area peptide (220 nm)
0.125	302.166
0.125	309.089
0.125	318.568
0.125	318.367
0.25	625.25
0.25	626.576
0.25	641.095
0.25	639.734
0.5	1226.29
0.5	1217.45
0.5	1275.67
0.5	1264.08
0.75	1845.06
0.75	1838.72
0.75	1963.99
0.75	1952.52

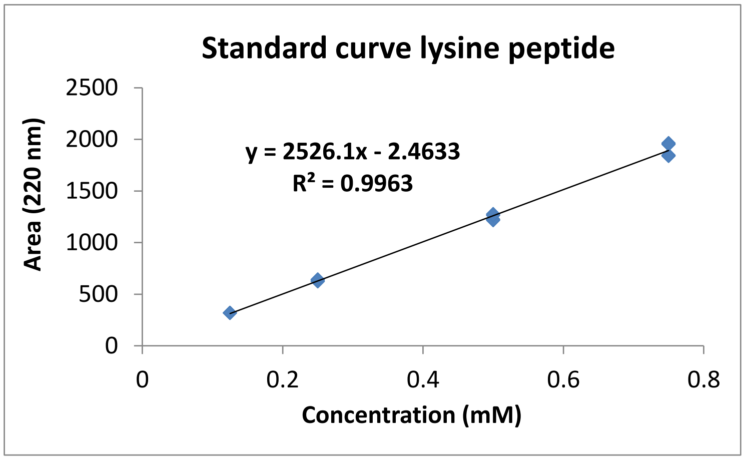

Figure S11: Standard curve for lysine peptide used in the experiments with FITC

The peak areas of the lysine peptide at 220 nm are plotted against the concentrations and the obtained *k, m* and R^2 values are shown.
